# Assessing the Prevalence and Histopathology of *Linguatula serrata* Infection of Camels and Cattle in Zabol County, Sistan and Baluchestan Province, Iran

**DOI:** 10.1002/vms3.70143

**Published:** 2024-11-27

**Authors:** Mohammad Rahnama, Tayebeh Bameri, Soheil Sadr, Narges Lotfalizadeh, Majid Alipour Eskandani, Javad Khedri

**Affiliations:** ^1^ Department of Food Hygiene and Quality Control, Faculty of Veterinary Medicine University of Zabol Zabol Iran; ^2^ Doctor of Veterinary Medicine Graduate Student University of Zabol Zabol Iran; ^3^ Department of Pathobiology, Faculty of Veterinary Medicine Ferdowsi University of Mashhad Mashhad Iran

**Keywords:** Camel, Cattle, Iran, *Linguatula serrata*, Parasite, Sistan and Balouchestan

## Abstract

**Background/objective:**

*Linguatula serrata* (*L. serrata*) is a worldwide zoonotic parasite and one of the most widespread pathogens. This study aimed to determine the prevalence of *L. serrata* in cattle and camels of Zabol County, Sistan and Baluchestan Province, Iran.

**Methods:**

From March 2014 to February 2021, 300 cattle (48 female and 252 male) and 300 camels (31 female and 269 male) were examined at the Zabol slaughterhouse, encompassing different age groups. The cattle breeds were indigenous (native Sistani breeds) and non‐indigenous (breeds introduced from external regions), while all the camels were native breeds. To identify *L. serrata* nymphs, three samples of mesenteric lymph nodes (MLNs) were meticulously collected in phosphate‐buffered saline immediately after slaughtering.

**Results:**

According to the results, 4.33% (13/300) of cattle were infected with *L. serrata*, with six females and seven males infected. There was a significant difference in the prevalence of infection between females (6/48, 12.5%) and males (7/252, 2.77%) of cattle (*p* = 0.009). Moreover, cattle older than 3 years had significantly more infections with *L. serrata* nymphs (16.07%, 9/56) than cattle of other ages (*p* < 0.001). Compared to other seasons, autumn showed a high prevalence of parasites (8.1%, 6/74) (*p* = 0.017). Regarding the camels, only 3.66% (11/300) were infected, and there was no significant difference in infection rates between male and female camels or between seasons (*p* > 0.05). The infection rate was higher in camels older than 3 years (n = 9) than in camels younger than 3 years (*p* < 0.05). The MLNs of infected cattle were edematous, red, enlarged and characterized by an eosinophilic granulomatous reaction with mononuclear inflammatory cells.

**Conclusion:**

The present study had low rates of *L. serrata* infection because the farms were clean, and cattle and camels did not have contact with final hosts such as dogs and wild canids. This suggests that their management strategies, including secure and preventative measures, were effective.

## Graphical abstract

This study conducted in southeast Iran aimed to assess the prevalence and pathology *of Linguatula serrata*, a zoonotic pathogen, in cattle and camels. The research involved 300 cattle and 300 camels sampled between 2014 and 2021. The findings indicated that 4.3% of cattle were infected with *L. serrata* nymphs, with a significantly higher prevalence in female cattle compared to males. Older cattle (above 3 years) had a higher infection rate than younger ones. The parasite had a higher prevalence in the autumn season. Overall, the study updates the prevalence of *L. serrata* in the region and highlights the need for continued vigilance regarding zoonotic transmission. Among 300 camels, only 11 (3.66%) were infected, with no significant differences observed in terms of gender, or season. However, camels more than 3 years old had a higher infection rate than camels under 3 years old.

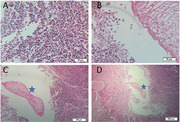

## 1 Introduction

*Linguatula serrata* (*L. serrata*) belongs to the phylum Pentastomida and the family Linguatulidae, a still‐enigmatic group of worm‐like which inhabit the respiratory system (nasal sinuses and nasopharynx) of carnivorous (Fard et al. 2010; Oryan et al. 2008; Raele et al. 2022). There are several intermediate hosts for *L. serrata* nymphs, including cattle, sheep, goats, horses, rabbits, wallabies and rodents (Barton et al. 2020; Barton et al. 2020; Hajipour and Tavassoli 2019). After releasing eggs, they are infectious because they contain fully developed larvae. Once the intermediate host ingests the eggs, the larvae hatch and move to different organs away from the intestines.

Dogs and carnivores as final hosts play a crucial role in the life cycle of the *L. serrata* (Attia et al. 2024). If the livestock‐keeping environment is well‐controlled and appropriate health measures are taken, parasite eggs can be prevented from entering the environment (Islam et al. 2018). For example, the cycle of parasite transmission can be interrupted by limiting the access of dogs and carnivores to animal husbandry environments and preventing them from feeding on raw and contaminated meat (Shamsi et al. 2017). Moreover, using anti‐parasitic drugs to treat livestock and pets can help reduce the rate of infection.

The parasite develops different stages of nymphs. For example, cows and goats have nymph stages that develop mainly in the mesenteric and hepatic lymph nodes, while horses and camels have nymphs mainly in the mesenteric lymph nodes (MLNs) (Abuseir 2021; Islam et al. 2018). In contrast, sheep have nymphs concentrated in the mediastinal nodes (Azizi, Nourani, and Moradi 2015). The life cycle of the organism *L. serrata* is complete once the final host consumes the infected viscera of the intermediate host. Humans are not only infected by ingesting nymphs from raw or undercooked viscera (i.e., liver, lungs, lymph nodes) from intermediate hosts, but they also serve as accidental hosts when ingesting eggs in contaminated water, vegetables and fruits (Sarmadian et al. 2021). The condition is called nasopharyngeal linguatulosis (halzoun syndrome) and visceral linguatulosis. The symptoms of nasopharyngeal linguatulosis in the final hosts include sneezing, coughing, lacrimation, coryza, yellow nasal discharge, facial edema, dyspnea, dysphagia, vomiting and frontal headache (Nagamori et al. 2019).

There have been several reports in various parts of Iran to determine the prevalence of *L. serrata* in camels, cattle, buffaloes, sheep, goats, dogs and humans (Alborzi, Molayan, and Akbari 2013; Dehkordi et al. 2014; Fard et al. 2012; Nematollahi et al. 2015; Tabaripour et al. 2017; Yazdani et al. 2014). According to recent studies, there are significant concerns regarding linguatulosis in Iran. It was reported that 15.84% of cattle in southeastern Iran were infected with *L. serrata*, illustrating the extent to which the parasite is prevalent (Mohammadi et al. 2020). Moreover, according to a study on camels examined in Isfahan Province, 21.12% had *L. serrata* nymphs (F. Rezaei, Tavassoli, and Javdani 2012).

In addition, a systematic review and meta‐analysis of linguatulosis prevalence across Iran revealed a high prevalence of the disease in the West Azerbaijan Province at 68%. A rate of 0.23% was recorded in Khuzestan Province by comparison (Tabaripour et al. 2019). The infection percentages ranged from 11.66% to 65.79% in slaughtered sheep in the Urmia and Isfahan provinces (Kheirabadi et al. 2015; Yakhchali et al. 2009). Furthermore, 11.5% of sheep in Shiraz had MLNs containing *L. serrata* (Shekarforoush, Razavi, and Izadi 2004). However, to the best of our knowledge about *L. serrata* prevalence in Zabol County has been void. Hence, the present study aimed to determine the prevalence of *L. serrata* nymphs in cattle and camels of Zabol County, Sistan and Baluchestan Province, Iran.

## 2 Materials and Methods

### 2.1 Study Area

Zabol County, Sistan and Baluchestan Province, Iran, was the site of the current study. In terms of latitude and longitude, it lies between 30°18′ and 31°20′ N, and 61°10′ and 61°50′ E. It covers 334 km^2^ and has 3,29,317 people living there. It is bordered by Afghanistan and Pakistan to the north and east, respectively. Zabol averages an elevation between 475 and 500 m above sea level and a relative humidity of 40%. During the year, the region's average maximum and minimum temperatures are 49°C and −8°C, respectively, while the average rainfall is 59 mm.

### 2.2 Sampling Procedure

There was a random sampling of 300 cattle (48 female and 252 male) and 300 camels (31 female and 269 male) from the Zabol slaughterhouse in March 2014 to February 2021 of different age groups (based on the eruption of permanent incisor teeth). Cattle were divided between indigenous breeds and those imported from other regions, while camels were all indigenous breeds. Upon arrival at the slaughterhouse, these animals were randomly selected based on their age, ranging from less than 1 year old to 1–2 years old, to 2–3 years of age, and those older than 3 years. In addition, a significant element of the study is its investigation of the husbandry practices adopted. No final hosts were present in the farms of the animals were belong, so cattle and camels were confined to areas devoid of final hosts. This allowed the environment to be relatively hygienic.

### 2.3 Parasitological Procedures

To determine whether *L. serrata* nymphs are present or not in the MLNs of each animal, three samples from MLNs were collected in PBS immediately after slaughtering (Islam et al. 2018; F. Rezaei, Tavassoli, and Mahmoudian 2011). These samples were then transferred to the Food Hygiene and Quality Control laboratory at the Veterinary Faculty of Zabol University, Iran, to examine further investigations. First, samples were cut into small pieces, immersed in a solution of normal saline (0.9% NaCl), and examined under a dissecting microscope to see if any *L. serrata* nymphs were present. Negative samples were digested in 200 mL of a digestive solution that contained 5 g of pepsin (7197, Merck) in 25 mL of 37% hydrochloric acid (317, Merck) in 1000 mL of distilled water and then incubated at 37°C for 24 h (H. Rezaei et al. 2012). A fine sieve was then used to wash the suspensions in tap water. Under a stereomicroscope, the nymphs were collected and counted. During the collection process, MLNs with nymphs were placed in a 10% formalin solution, a fixative solution (Alborzi, Molayan, and Akbari 2013).

### 2.4 Histopathological Examination

All MLNs with immature stages of *L. serrata* were fixed in 10% neutral buffered formalin for 72 h, then dehydrated in graded ethylalcohol to prepare fine paraffin wax blocks, and sections were cut into 5‐mm sections using a rotary microtome. Haematoxylin and eosin staining were applied to evaluate the sections under a light microscope.

### 2.5 Statistical Analysis

Stata software, version 11.2, was used to analyse the data. Various tests were carried out, including Pearson chi‐squared, likelihood ratio of Chi‐squared, linear by linear association chi‐squared and Fisher's exact test. Descriptive statistics were used with 95% confidence intervals to analyse qualitative data. Significant results were determined at *p *< 0.05.

## 3 Results

**FIGURE 1:**
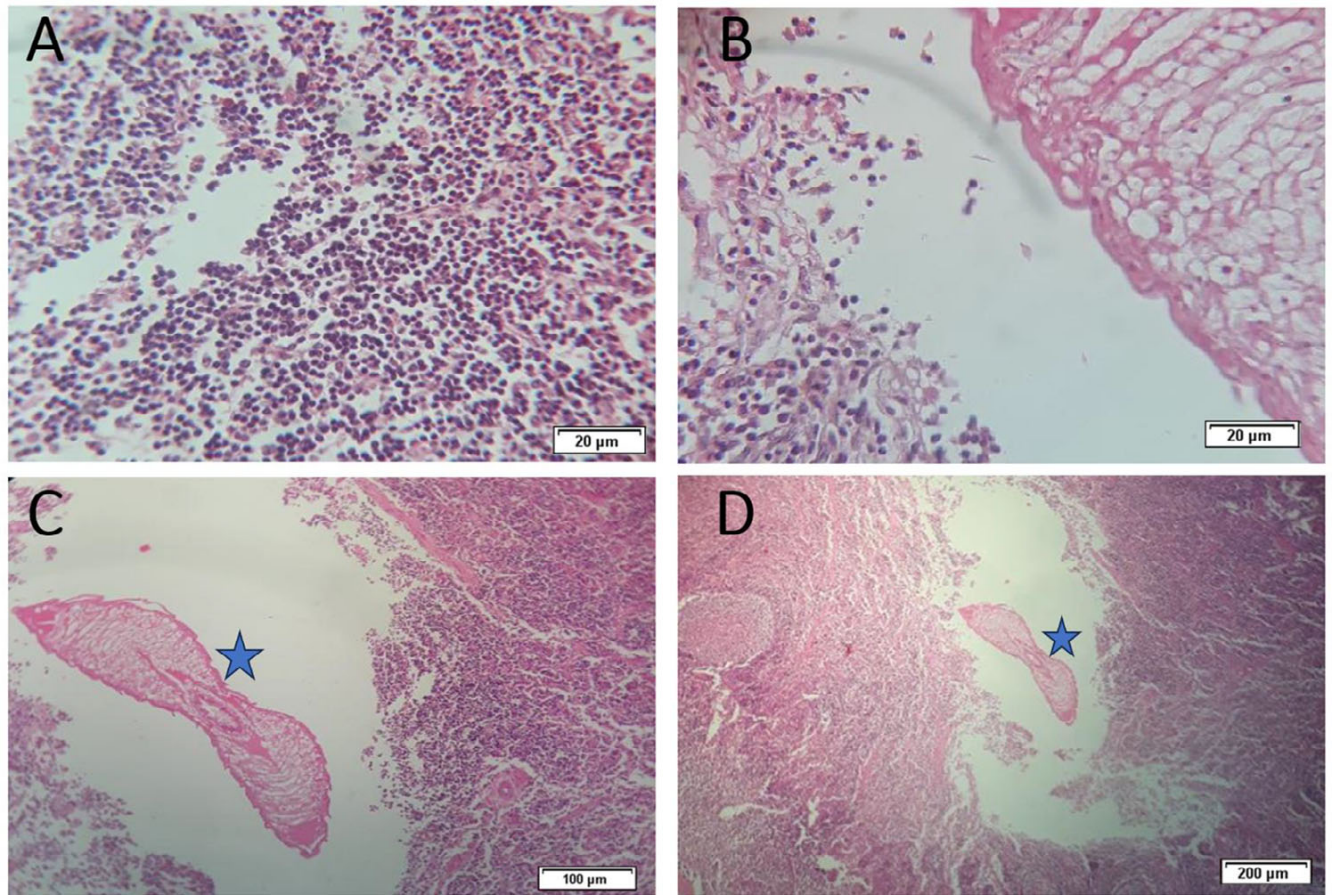
A, B) Granulomatous reactions were observed with giant cells, lymphocytes, eosinophils and macrophages at the parasite degeneration site. (C, D) The blue stars are *Linguatula serrata* nymphs.

Based on the results of the study, 4.33% (13/300) of cattle were infected with *L. serrata*, with six females and seven males infected. There was a significant difference in the prevalence of infection between females (6/48, 12.5%) and males (7/252, 2.77%) of cattle (*p* = 0.009). Moreover, cattle older than 3 years had a significant higher infection rate with *L. serrata* nymphs (16.07%, 9/56) than cattle of other ages (*p* < 0.001). No significant difference was observed among indigenous and non‐indigenous cattle infection (*p *> 0.05). Furthermore, it was found that the highest prevalence of infection occurred in the autumn (8.1%, 6/74) and it was significantly higher compared with the other seasons (*p* = 0.017) (Tables 1, 2, 3, 4). The MLNs of infected cattle were edematous, red, enlarged and characterized by an eosinophilic granulomatous reaction with mononuclear inflammatory cells. At the parasite degeneration site, granulomatous reactions were observed with giant cells, lymphocytes, eosinophils and macrophages (Figure 1).

**TABLE 1:** Prevalence and characterization of infected cows based on breed.

Breed	Total number of cows	Infected cows	Infection prevalence %	Analysis test	*p* value
Indigenous	150	5	3.33	Pearson chi‐square	0.395
Non‐indigenous	150	8	5.33		

**TABLE 2:** Prevalence and characterization of infected cows based on seasons.

Seasons	Total number of cows	Infected cows	Infection prevalence %	Analysis test	*p* value
Autumn	74	6	8.10	Likelihood ratio of chi‐square	0.017
Spring	74	0	0		
Summer	78	2	2.56		
Winter	74	5	6.75		

**TABLE 3:** Prevalence and characterization of infected cows based on age.

Age	Total number of cows	Infected cows	Infection prevalence %	Analysis test	*p* value
< 1	31	0	0	Linear by linear association chi‐square	< 0.001
1–< 2	70	0	0		
2–< 3	143	4	2.79		
> 3	56	9	16.07		

**TABLE 4:** Prevalence and characterization of infected cows based on gender.

Gender	Total number of cows	Infected cows	Infection prevalence %	Analysis test	*p* value
**Male**	252	7	2.77	Fisher's exact test	0.009
**Female**	48	6	12.5		

**FIGURE 2:**
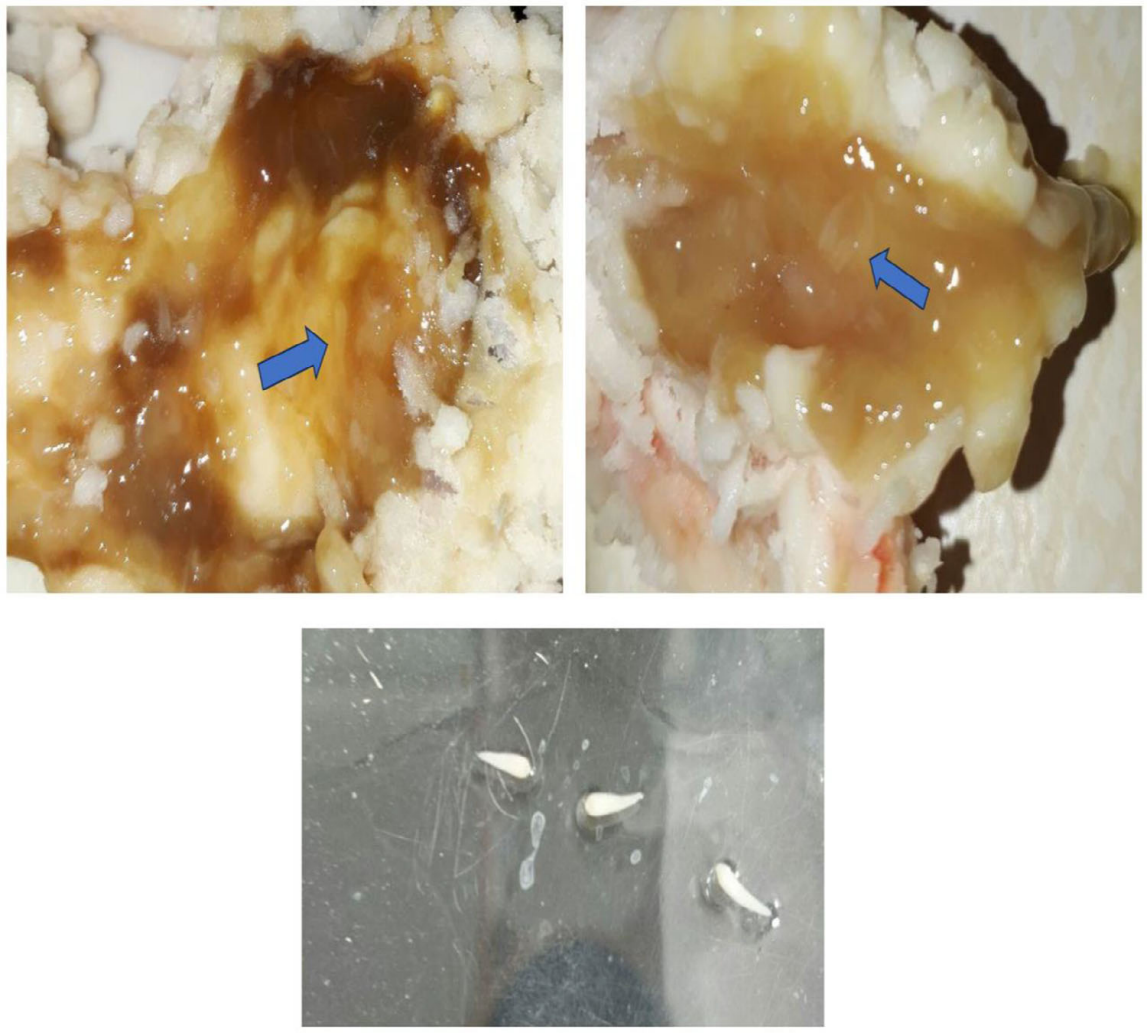
Macroscopic examination of the lymph nodes in the infected camels. The blue arrows are *Linguatula serrata* nymphs.

A total of 300 camels were examined and only 3.66% (11/300%) of them were infected. There were no significant difference in terms of sex or season (*p *> 0.05) among the infected camels. However, the infection rate of camels older than 3 years was significantly higher (n = 9) than camels younger than 3 years (*p *< 0.05) (Figure 2).

## 4 Discussion

As definitive hosts, dogs and carnivores play a crucial role in the life cycle of *L. serrata*. To reduce the spread of infection, controlling the livestock‐keeping environment and adopting sanitary measures is necessary (Fard et al. 2012). For example, limiting the access of dogs and carnivores to animal husbandry environments and preventing them from feeding on raw and contaminated meat can interrupt the parasite transmission cycle (Sarmadian et al. 2021). Furthermore, using anti‐parasitic drugs to treat livestock and pets helps reduce the infestation rate. In this way, by carefully controlling the environment and reducing the contact of livestock with the final hosts, it is possible to prevent the spread of the *L. serrata* parasite and ensure the health of livestock and humans (Sazmand, Joachim, and Otranto 2019).

According to the present study, 4.33% prevalence of *L. serrata* nymph infection in cattle was recorded, which was less than 14.8% in Babol (Youssefi and Moalem 2010), 16.1% in Kerman (Nourollahi Fard et al. 2011), 18.9% in Tabriz (Nematollahi et al. 2015), 12.8% in Kermanshah (Hashemnia et al. 2018) and 19% in India (Ravindran et al. 2008). The variation in the prevalence of *L. serrata* infection can be related to types of animals, sample size, parasite distribution and management systems. The temperature and humidity of the environment may thus play a critical role in the epidemiology of this infection. A significant difference was observed between the prevalence of *L. serrata* nymph infection in female cattle and that of male cattle in this study. This finding is similar to a survey by Dehkordi et al. (2014), who reported the prevalence of *L. serrata* nymphs to be significantly higher in females than in males (Dehkordi et al. 2014). Among the grazing systems, slaughtering, management and grazing of livestock systems, there is a difference in infection rates between the sexes, mainly due to the higher mean age of females than males. A high infection rate of *L. serrata* nymphs was found in the current study on older cattle, consistent with previous studies, which found that the highest infection rate was found in cattle older than 3 years old. In young groups, the prevalence of this disease is less common, which may be due to different feeding practices (milk instead of grass) and relatively fewer encounters with the definite host. Neither the prevalence of the disease among indigenous cattle nor that of non‐indigenous cattle was found to differ significantly.

Eleven camels (3.66%) were infected out of 300 studied. A noteworthy finding of the study was that sex and season had no statistically significant effects on camel infection rates. Nevertheless, there was a higher infection rate among camels over 3 years old than those under 3. As a consequence of this observation, it is interesting to see how the infection rate among camels has remained remarkably consistent across diverse demographic categories within the population, suggesting the particular infection under study has a uniformly low prevalence throughout the herd. Based on these results, public and veterinary health efforts can be used to develop new strategies for managing diseases among camel populations. This illustrates the need for further research into factors influencing low infection rates among camel populations. The current study showed that the highest infection rate of the *L. serrata* nymph was recorded in autumn, and the lowest was observed in spring. This result align with previous studies (Esmaeilnejad et al. 2017; Gharekhani et al. 2017). In the present study, the histopathological changes observed in the MLNs included hyperemia, haemorrhage, necrosis, edema, swelling, softening, redness and granulomatous reactions characterized by infiltration of mononuclear inflammatory cells, eosinophils and macrophages around the parasite.

No previous research has been examined in the particular geographical scope of the present study, which signifies a pioneering effort in an area where there has been no previous investigation. An arid climate and extreme temperatures characterize this area of Iran, and this climate is noticeable, unlike any other area in Iran, due to its high temperatures and aridity. This aridity plays a pivotal role in inhibiting the complete life cycle of parasites, thereby contributing to a reduced prevalence and infection rate. Furthermore, the husbandry practices employed are a noteworthy facet of the present study. The current study's comprehensive examination encompassed both mesenteric and hepatic lymph nodes. No positive samples were found in the hepatic lymph nodes, but MLNs exhibited contamination exclusively. According to the study findings, older females have higher infection levels in autumn. This intriguing pattern warrants careful consideration and further research in the context of public health and prevention management strategies for disease prevention and control. Based on these patterns, it is possible to tailor health policies to focus on targeted interventions targeting older female individuals in both host groups during the autumn season to maximise their health.

## 5 Conclusion

In conclusion, the results of the present study update the prevalence of *L. serrata* nymphs in cattle and camels in southeast Iran. Although the results of the present study represent relatively low *L. serrata* infections in these animals, there remains a potential risk of human infection. Furthermore, public health education, avoiding raw MLNs in dogs, and changing food habits (not eating raw viscera) can reduce the infection. In general, further investigations into both domestic and wild herbivores and carnivores, together with more detailed studies on the occurrence of this infection in humans, are suggested.

## Author Contributions

**Mohammad Rahnama**: conceptualization, data curation, formal analysis, investigation, methodology, resources, software, validation, visualization, writing–original draft, writing–review and editing. **Tayebeh Bameri**: data curation, formal analysis, investigation, methodology, writing–review and editing. **Soheil Sadr**: Conceptualization, formal analysis, investigation, methodology, project administration, supervision, validation, visualization, writing–original draft, writing–review and editing. **Narges Lotfalizadeh**: data curation, investigation, methodology, software, validation, writing–review and editing. **Majid Alipour Eskandani**: conceptualization, data curation, formal analysis, investigation, methodology, resources, software, validation, visualization, writing–original draft. **Javad Khedri**: conceptualization, data curation, formal analysis, investigation, methodology, resources, software, validation, visualization.

## Ethics Statement

All applicable international, national and/or institutional guidelines for the care and use of animals were followed. The study methods have been approved the study procedure by the ethical committee of the Animal Welfare Committee at Zabol University IR.UOZ.REC.1402.014.

## Conflicts of Interest

The authors declare no conflicts of interest.

## Consent

The authors have nothing to report.

### Peer Review

The peer review history for this article is available at https://publons.com/publon/10.1002/vms3.70143.

## Data Availability

The datasets generated during and/or analysed during the current study are available from the corresponding author upon reasonable request.
